# Struggling or Liberating? The Effects of Machiavellianism on Break-Up Distress

**DOI:** 10.3390/ijerph192114581

**Published:** 2022-11-07

**Authors:** Peiyan Zeng, Wenjing Jin, Yibo Shi, Wanying Hu, Yaoguo Geng, Tingting Zhan

**Affiliations:** School of Marxism, Zhengzhou University, Zhengzhou 450001, China

**Keywords:** Machiavellianism, break-up distress, psychological capital, gender difference

## Abstract

Negative emotions caused by break-up are the key work of university students’ psychological intervention. It is important to explore the specific factors of break-up distress for university students’ psychological intervention. Therefore, we investigated 869 university students to examine the effect of Machiavellianism and psychological capital on break-up distress, as well as its gender difference. The results indicated that high Machiavellians experience more break-up distress. Moreover, through structural equation models, we found that as for female university students, psychological capital mediated the relationship between Machiavellianism and break-up distress. However, as for male university students, the mediation effect was not significant. It means that for female university students, psychological capital acted as the mechanism to connect Machiavellianism and break-up distress.

## 1. Introduction

The dissolution of romantic relationships is one of the most distressing experiences for adolescents and early adulthood individuals and has implications for life satisfaction and mental health [1,2,3,4]. Zhang classified the psychological counseling of 207 university students and found that nearly 40% of the university students came for counseling because of love and emotional problems [5]. Therefore, it is an important issue to explore the antecedents of break-up distress and its psychological mechanism beneath the relationship mentioned above.

Break-up distress consists of a series of psychological and physical disturbances after the romantic relationships end, such as guilt, anxiety, anger, grief, depression, sleep problems, and eating problems [6]. Previous studies have highlighted that many factors act on break-up distress, such as the duration and end of romantic relationships, attachment types, and personality traits [2,7,8]. For example, Waller and MacDonald found that self-esteem was strongly associated with break-up distress: low self-esteem individuals experience more distress than others when rejected by their partner [9]. Tashiro and Frazier investigated the effect of the Big Five on break-up distress, and they found that neuroticism was positively correlated with break-up distress [10]. Recent research has revealed that to the Big Five, break-up distress is also associated with some dark personality traits. Moroz et al. have highlighted that Machiavellianism and psychopathy were positively correlated with their break-up distress [11].

Machiavellianism was proposed by the Italian political scientist and historian Machiavelli. As a synonym for deception, manipulation, and unscrupulous means, Machiavellianism is included in the “dark triad” [12,13]. High Machiavellians are usually cynical, have low levels of empathy, enjoy controlling others, and often manifest as manipulative and deceitful partners in romantic relationships [14]. The dissolution of romantic relationships deprives high Machiavellians of the object they were manipulating, so they will experience distress when confronted with the break-up, especially when they ended the relationship passively [11]. Bereczkei highlighted that due to the existence of alexithymia, high Machiavellians seem to be emotionally cold, but in fact have intense internal emotional experiences. Thus, high Machiavellians appear to be lighthearted, but still report high levels of break-up distress [15]. Moreover, the “Innocent Darkness” framework states that high Machiavellians may not understand the degree of harm their actions cause to others, so the dissolution of romantic relationships may leave high Machiavellians more puzzled and angrier [16,17,18]. Additionally, Birkás has noted that high Machiavellians report higher levels of anxiety and sensitivity to rejection when they lose a manipulative object [19]. Based on the previous studies above, we propose:

**Hypothesis** **1.** *Machiavellianism was positively correlated with break-up distress.*

Unfortunately, research on Machiavellianism and break-up distress is rather limited. Some researchers mainly focused on the role of Machiavellianism in break-up distress, but few studies have investigated its internal impact mechanism. Based on previous studies, psychological capital may play an important role between Machiavellianism and break-up distress. Psychological capital refers to the optimism, hope, resilience, and self-efficacy that individuals exhibit as they grow [20]. Studies have revealed that personality is a powerful factor which is closely related to psychological capital, especially the Big Five [21,22]. Some researchers found that extraversion, openness, and agreeableness were positively associated with psychological capital [22]. However, Machiavellianism, as part of the dark personality, has less research on psychological capital. A longitudinal study based on Chinese university students demonstrated that Machiavellianism negatively predicted psychological capital [23]. Therefore, the present study selected Machiavellianism as a personality variable to verify the effect of personality on psychological capital.

Recent empirical studies have shown that, as a kind of psychological capital which has an important impact on individuals’ production and life, resilience is a strong influencing factor of individuals’ break-up distress. Some researchers have highlighted that individuals who scored higher on resilience would experience less depression than others when a romantic relationship ends [24]. Furthermore, individuals with higher levels of psychological capital can be more optimistic, flexible, and adaptive in coping with negative life events [25], so they would experience less distress when a romantic relationship ends. As mentioned above, we have reason to propose:

**Hypothesis** **2.** *Psychological capital mediates the relationship between Machiavellianism and break-up distress.*

In reviewing the literature, Machiavellianism, psychological capital, and break-up distress all vary by gender. Semenyna and Honey highlighted that males scored higher than females on Machiavellianism [26]. In addition, Jonason and Davis proposed that the level of Machiavellianism in males is significantly higher than that in females, which is manifested in more interpersonal manipulation and less empathy [27]. Furthermore, Zhu et al. reported the cross-cultural consistency of gender differences [28].

Regarding the effects of gender on psychological capital, some studies have highlighted that males score higher on psychological capital than women [29,30]. However, contrary to these results, some researchers had different findings. Singh and Garg believed that females have higher levels of psychological capital. When faced with troubles, females are calmer and more in control than males [31]. Moreover, females are more willing to fully mobilize personal resources and proactively seek help to better cope with challenges [32].

Similarly, gender differences in break-up distress have been controversial. Some studies have shown that females have significantly higher levels of break-up distress than males [6,33,34]. Compared with males, females experience more negative emotions, such as sadness and fear, after a romantic relationship ends. Additionally, Shulman et al. found that females experience more distress after a romantic relationship ends, which will influence the quality of future romantic relationships [35]. However, Knox et al. and Carter et al. believed that males have higher levels of break-up distress than females [36,37]. Moreover, Hill et al. found that females are more likely to end their romantic relationships on their own initiative. Facing the sudden end of a romantic relationship, boys experience more negative emotions, such as confusion, remorse, anger, etc., which means higher levels of break-up distress [38]. To sum up, although some of the research conclusions are slightly different, it still points out that males and females perform differently in Machiavellianism, psychological capital, and break-up distress. Based on this, we can propose:

**Hypothesis** **3.** *There would be gender differences in the mediation model.*

**Figure 1:**
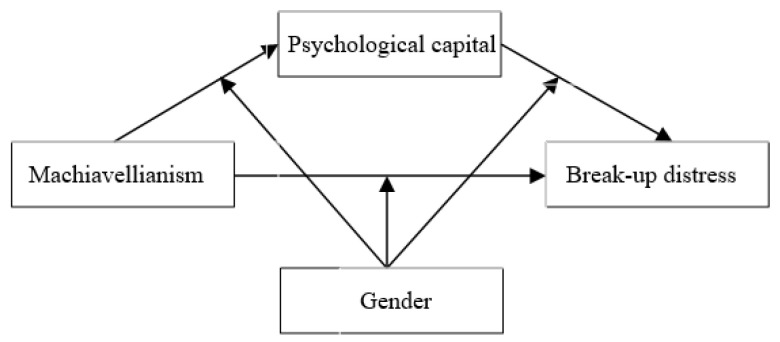
The hypothetical theoretical model.

The present study aimed to determine whether psychological capital mediates the relationship between Machiavellianism and break-up distress. Furthermore, we determined whether gender moderates the mediation model mentioned above. The hypothetical theoretical model is as follows (Figure 1).

## 2. Methods

### 2.1. Participants

Using the snowball sampling method, students from two universities in Zhengzhou were asked if they have experienced a break-up in the past six months. A total of 600 university students were reserved in the present study and continued the subsequent research. In total, 563 valid questionnaires were finally collected, with an efficiency was 93.8%. Among them, 285 were male and 278 were female. Participants ranged in age from 16 to 23, with a mean age of 19.61 (*SD* = 1.20) years. All procedures involving human participants in the present study were in compliance with the ethical standards of the institution.

### 2.2. Measures

The Machiavellianism Personality Inventory (MPS) was composed of 16 items [39], including four subscales: “distrust of others”, “amoral manipulation”, “desire for control”, and “desire for status”. The scale was rated on a 5-point Likert scale (1 = “strongly disagree”, 5 = “strongly agree”), with higher scores representing higher levels of Machiavellianism. The Cronbach’s α for the total scale in the present study was 0.859.

The Compound PsyCap Scale (CPC-12) was composed of 12 items and included four subscales [40]: “hope”, “optimism”, “resilience”, and “self-efficacy”. The scale was scored on a 6-point Likert scale (1 = “strongly disagree”, 6 = “strongly agree”), with higher scores representing higher levels of psychological capital. The Cronbach’s α for the total scale in the present study was 0.933.

The Break-up Distress Scale was composed of 16 items [6]. The scale was scored on a 4-point Likert scale (1 = “not at all”, 4 = “very much so”), with higher scores representing higher levels of break-up distress. The Cronbach’s α for the total scale in the present study was 0.978. 

### 2.3. Statistical Analyses

Firstly, SPSS 21.0 was utilized to execute descriptive statistics and correlation analysis. Then, PROCESS macro for SPSS was utilized to test the moderated mediation model. To determine whether the indirect and direct effects were statistically significant, the bias-corrected bootstrapping method (5000 resamples) was conducted via Process to test the 95% confidence interval of indirect effects. We examined whether psychological capital mediated the relationship between Machiavellianism and break-up distress by using Model 4 and tested whether the mediation model was moderated by gender using Model 59. Finally, Amos 21.0 was used to conduct the multi-group analysis.

## 3. Results

### 3.1. Descriptive Statistics and Correlation Analysis

Males scored significantly higher on Machiavellianism but scored significantly lower on psychological capital than females. Moreover, they experienced more distress from the break-up. Machiavellianism was correlated with psychological capital negatively, while the correlation between Machiavellianism and break-up distress was positive. Psychological capital was negatively correlated with break-up distress (see Table 1). Hypothesis 1 was supported.

### 3.2. The Mediating Role of Psychological Capital

The results demonstrated that Machiavellianism was negatively associated with psychological capital (*a* = −0.17, *SE* = 0.04, *p* < 0.01). When Machiavellianism and psychological capital entered the regression equation at the same time, Machiavellianism was positively associated with break-up distress (*c’* = 0.20, *SE* = 0.05, *p* < 0.01), while psychological capital was negatively associated with break-up distress (*b* = −0.15, *SE* = 0.05, *p* < 0.01). The results illustrated that the mediating effect of psychological capital between Machiavellianism and break-up distress was significant, *ab* = 0.02, *Boot SE* = 0.02, with 95% *CI* [0.01, 0.04]. Hypothesis 2 was supported.

### 3.3. The Moderating Role of Gender

The results showed that psychological capital mediated the relationship between Machiavellianism and break-up distress. Then, model 59 was used to test the moderating role of gender in the mediation model. In the present study, two equations were examined, Equation (1) examined the role of Machiavellianism on psychological capital and the moderating role of gender, and Equation (2) examined the roles of Machiavellianism and psychological capital on break-up distress, then test whether gender played a moderating role or not. Meantime, gender was treated as a dummy variable (1 for male and 0 for female) and other continuous variables were standardized. The results showed that the effect of the interaction term (Machiavellianism × gender) on break-up distress was significant (see Table 2). Hypothesis 3 was supported.

We used AMOS 21.0 to test the mediating role of psychological capital and its gender differences clearly. Firstly, the mediation model was tested separately according to gender. The results showed that the fitted indicators for males were: *χ*^2^/*df* = 2.20, *TLI* = 0.98, *NFI* = 0.97, *CFI* = 0.98, *RMSEA* = 0.07, while the fitted indicators for females were: *χ*^2^/*df* = 2.92, *TLI* = 0.96, *NFI* = 0.95, *CFI* = 0.96, *RMSEA* = 0.08. The fit indices mentioned above were all acceptable, then the multi-group analysis could be conducted. M1 was set as the unrestricted model, and M2 was set as the model with all equal structural coefficients, with the following fit indices for the models (see Table 3). The results showed that: Δ*χ*^2^ = 8.56, Δ*df* = 3, *p* < 0.05, which indicates there were significant differences between the unrestricted model (M1) and the model with all equal structural coefficients (M2).

**Figure 2:**
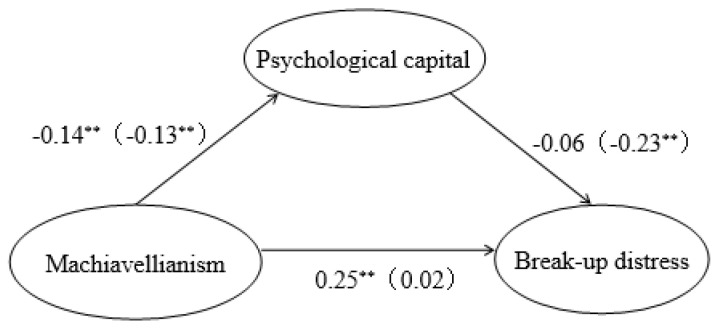
Gender differences in the mediating model (the coefficients outside the brackets were males while those inside the brackets were females). ** *p* < 0.01.

Figure 2 shows the standardized path coefficients for the model across gender. In the male group, Machiavellianism was positively associated with break-up distress (*β* = 0.25, *p* < 0.01). Machiavellianism was negatively associated with psychological capital (*β* = −0.14, *p* < 0.01); however, the effect of psychological capital on break-up distress was not significant (*β* = −0.06, *p* = 0.55). It is not sufficient to support the mediating role of psychological capital between Machiavellianism and break-up distress in the male group.

For female university students, the effect of Machiavellianism on break-up distress was not significant (*β* = 0.02, *p* = 0.63). Furthermore, Machiavellianism was negatively associated with psychological capital (*β* = −0.13, *p* < 0.01), and psychological capital was negatively associated with break-up distress. The results showed that for female university students, psychological capital completely mediated the relationship between Machiavellianism and break-up distress.

## 4. Discussion

The present study indicated the effects of Machiavellianism on break-up distress, as well as its underlying mechanism. On the one hand, our study elucidated the mediating role of psychological capital. On the other hand, it proposed the moderating role of gender. For female university students, psychological capital completely mediated the relationship between Machiavellianism and break-up distress, while for male university students, the mediation effect is not significant. The theoretical importance and the relevant practical value of results on the psychological intervention of university students are prominent.

As predicted, we found that high Machiavellians experienced more break-up distress. There is some inconsistency between this conclusion and previous research [11,16]. For this result, we have some explanations. Firstly, Machiavellianism acts as an “amplifier” when individuals face negative life events [41], which makes high Machiavellians usually experience more negative emotions, such as anxiety, depression, etc. [42]. So, they will pay more attention to their negative emotions after the romantic relationships end, which may lead them to report higher break-up distress. Additionally, Birkás highlighted that Machiavellianism is positively related to the past negative time perspective, that is, high Machiavellians tend to pay more attention to their negative past experiences [43]. The dissolution of romantic relationships is one of the major setbacks in personal relationships, and its impact can be far-reaching. Based on this, High Machiavellians will report higher levels of distress when they look back on the experience. Furthermore, high Machiavellians are keen on intrigue and manipulation, manifested as deceptive tactics in romantic relationships [14,44]. As a result, high Machiavellians reported greater distress when asked about break-up experiences. Finally, the positive correlation may also stem from lower self-esteem levels. Studies have demonstrated that high Machiavellians have lower levels of self-esteem [45], and low self-esteem puts more blame on themselves, so they will take a greater hit when a romantic relationship ends. Taken together, the dissolution of romantic relationships can cause more distress for high Machiavellians.

As hypothesized, we provided that psychological capital partially mediated the relationship between Machiavellianism and break-up distress. Machiavellianism, as one of the dark personality traits, has been reported to be associated with more internalization and externalization problems, such as aggression, eating problems, etc. [46,47]. We found that Machiavellianism was negatively associated with psychological capital, which is consistent with previous research [23]. High Machiavellians have lower levels of resilience and less positive emotions [48,49], and resilience and optimism are important components of psychological capital [20]. Consequently, high Machiavellians have less psychological capital than others. Moreover, the present study highlighted that psychological capital was one of the negative factors of break-up distress, which is supported by some previous research. It is generally known that higher levels of psychological capital mean higher levels of resilience and self-efficacy, as well as a more optimistic attitude and more hope in the face of setbacks [50,51,52]. Thence, individuals with high psychological capital can adapt better when faced with negative events, so they experience less distress with going through a break-up. In general, high Machiavellians experience more stress and negative emotions when they experience negative life events, and often hold a pessimistic attitude towards life, which means lower levels of psychological capital than others. As one of the major negative life events, the ending of romantic relationships has terrible effects on university students’ mental and physical health. When a romantic relationship ends, high Machiavellians inevitably perceive more distress due to their lower levels of psychological capital.

However, there were significant differences among groups of different genders. In detail, psychological capital completely mediated the effect of Machiavellianism on break-up distress among female university students, while the mediating effect is not significant in male university students. We believed that this gender difference is more likely to stem from the gender difference of Machiavellianism. Jonason (2018) proposed that the characteristics of Machiavellianism are more consistent with males, that is, Machiavellianism is better reflected in males [27]. For this reason, among male university students, Machiavellianism might have a higher coefficient for break-up distress. This conclusion, that the direct path coefficient of male university students is higher than that of female university students, is consistent with Geng et al. [16]. That is to say, after experiencing the dissolution of romantic relationships, high Machiavellian females are more flexible to change their perceptions and accept the facts, while males are more prone to rumination and experience more distress from the break-up. Females with high levels of Machiavellianism have a lower level of psychological capital, so they view things more negatively, and they have more negative emotions when a romantic relationship ends.

The present study explored the behaviors of high Machiavellians after the end of romantic relationships and enriched the research on Machiavellianism and break-up distress. Moreover, the present study focused on the influence of Machiavellianism and psychological capital on break-up distress, which provided solutions to emotional and behavioral problems caused by break-up from the perspective of personality traits and individual differences. Furthermore, our results have certain practical significance for psychological crisis intervention in universities. For female university students who are suffering from break-up distress, interventions on their psychological capital can be performed according to the suggestions of Luthans et al. [53], such as setting future goals, expressing positive attention to them, helping them make positive attributions, etc. In addition, psychological counseling workers in universities can use positive psychology group counseling technology, let students observe, experience and feel in group activities, so as to cultivate positive emotions and coping styles, then reduce break-up distress. Owing to the role of Machiavellianism in the break-up, it is necessary to pay close attention to high Machiavellians to reduce the possibility of crisis events when screening university students for mental health.

Despite the contributions of our findings, the study has several limitations inevitably. First of all, we did not examine the role of the break-up initiator. Some studies have highlighted that the perceived distress level of the one who ended the relationship is significantly weaker than that of the one who ended the relationship passively. This is an important issue for future research to incorporate break-up initiator into the model. Another important limitation to consider is that we only examined the role of Machiavellianism in the break-up. As an indispensable component of the dark triad, Machiavellianism is known as the “Malicious Two” with psychopathology [54,55]. Further studies, which take psychopathology and narcissism into account, will need to be undertaken. Furthermore, this study focused on the distress of the end of an unmarried romantic relationship and does not investigate the dissolution of a marital relationship (i.e., divorce). Whether there is any difference in the distress caused by the end of these two romantic relationships should be discussed in future research.

## 5. Conclusions

The present study showed that male university students and high Machiavellians suffered more from break-ups. In general, psychological capital acted as a mediating role in the relationship between Machiavellianism and break-up distress. Interestingly, the mediating effect was significant in female university students but not in male university students. The result suggested that strengthening psychological capital could be considered when dealing with campus cases related to female university students’ break-up distress.

## Figures and Tables

**Table 1:** Descriptive statistics and correlation analysis.

Variables	*M*	*SD*	1	2	3
1 Gender	—	—	—		
2 Machiavellianism	39.45	9.56	0.12	—	
3 Psychological capital	53.13	9.86	−0.14	−0.16	—
4 Break-up distress	25.85	11.85	0.35	0.18	−0.15

Note. All correlations were significant (*p* < 0.01).

**Table 2:** Moderated mediating effect analyses.

Variables	Equation (1): Psychological Capital			Equation (2): Break-Up Distress		
	*SE*	*β*	*t*	*SE*	*β*	*t*
Age	0.04	0.02	0.48	0.04	0.02	0.47
Machiavellianism	0.06	−0.16	−2.59 **	0.06	0.02	0.36
Gender	0.09	−0.25	−2.79 **	0.09	0.62	7.05 **
Machiavellianism × Gender	0.09	0.02	0.19	0.08	0.21	2.52 *
Psychological capital				0.07	−0.17	−2.53 *
Psychological capital × Gender				0.08	0.13	1.54
*R* ^2^	0.04			0.16		
*F*	6.00 **			17.31 **		

Note. * *p* < 0.05, ** *p* < 0.01.

**Table 3:** Fit indices for the two groups.

Model	*χ*^2^/*df*	TLI	CFI	RMSEA
M1	2.56	0.96	0.97	0.05
M2	2.57	0.96	0.97	0.05

## Data Availability

The data presented in this research are available on request from the corresponding author.
